# Proteomic and Bioinformatic Analysis of Decellularized Pancreatic Extracellular Matrices

**DOI:** 10.3390/molecules26216740

**Published:** 2021-11-08

**Authors:** Ming Hu, Huanjing Bi, Deana Moffat, Margaret Blystone, Lillian DeCostanza, Tchilabalo Alayi, Kaiming Ye, Yetrib Hathout, Sha Jin

**Affiliations:** 1 Department of Biomedical Engineering, Thomas J. Watson College of Engineering and Applied Sciences, Binghamton University, Binghamton, NY 13902, USA; mhu25@binghamton.edu (M.H.); hbi1@binghamton.edu (H.B.); dmoffat1@binghamton.edu (D.M.); mblysto1@binghamton.edu (M.B.); pdecost1@binghamton.edu (L.D.); kye@binghamton.edu (K.Y.); 2 Department of Pharmaceutical Sciences, School of Pharmacy and Pharmaceutical Sciences, Binghamton University, Binghamton, NY 13902, USA; talayi@binghamton.edu (T.A.); yhathout@binghamton.edu (Y.H.); 3 Center of Biomanufacturing for Regenerative Medicine, Binghamton University, Binghamton, NY 13902, USA

**Keywords:** pancreatic extracellular matrix proteins, proteomics, bioinformatics, decellularization, age and gender, KEGG pathway

## Abstract

Tissue microenvironments are rich in signaling molecules. However, factors in the tissue matrix that can serve as tissue-specific cues for engineering pancreatic tissues have not been thoroughly identified. In this study, we performed a comprehensive proteomic analysis of porcine decellularized pancreatic extracellular matrix (dpECM). By profiling dpECM collected from subjects of different ages and genders, we showed that the detergent-free decellularization method developed in this study permits the preservation of approximately 62.4% more proteins than a detergent-based method. In addition, we demonstrated that dpECM prepared from young pigs contained approximately 68.5% more extracellular matrix proteins than those prepared from adult pigs. Furthermore, we categorized dpECM proteins by biological process, molecular function, and cellular component through gene ontology analysis. Our study results also suggested that the protein composition of dpECM is significantly different between male and female animals while a KEGG enrichment pathway analysis revealed that dpECM protein profiling varies significantly depending on age. This study provides the proteome of pancreatic decellularized ECM in different animal ages and genders, which will help identify the bioactive molecules that are pivotal in creating tissue-specific cues for engineering tissues in vitro.

## 1. Introduction

Tissue matrix is a natural, bioactive material that provides unique microenvironments for tissue and organ formation and function. It is a complex network of hydrated macromolecular proteins and polysaccharides. It contributes not only mechanical support, but also physiochemical cues to regulate tissue structure and cell fate [1,2,3,4]. Decellularized pancreata, for instance, have been used widely as three-dimensional scaffolds to support the proliferation of repopulated islet cells or the differentiation of stem cells into pancreatic lineages [5,6,7,8]. We have recently reported that decellularized pancreatic extracellular matrix (dpECM) prepared from rat pancreata can enhance human pancreatic tissue development during human pluripotent stem cell pancreatic differentiation with both induced pluripotent stem cells (iPSCs) and embryonic stem cells (hESCs). This phenomenon suggests the presence of tissue-specific cues for pancreatic development within the dpECM [9]. Proteomic analysis of dpECM led to the discovery of the instructive function of type V collagen in inducing the self-assembly of iPSC-derived endocrine cell clusters into functional islets [10]. Proteomic analysis also helped identify signaling molecules from dpECM profiling that can stimulate the maturation of pancreatic islet organoids developed from stem cells [11]. While dpECM is rich in signaling molecules, identifying the proteins that can serve as tissue-specific cues for engineering pancreatic tissue remains largely uncompleted.

It has been reported that aging of the dermis alters both the ingredient content of proteins in the ECM and the structure of the protein networks [12]. The aging of vascular ECM results in the fragmentation of some large ECM proteins, such as elastin and collagen [13]. Interestingly, studies in both human and rodent unveiled postnatal development of pancreatic islets after birth [14,15]. A line of evidence suggested that neonatal islet cells proliferate rapidly before the age of 2 [16]. Such postnatal acquisition of functional islets hints at unique signals in newborn tissue/organ microenvironments [14,17]. These studies imply that dpECM prepared from young animals may contain strong instructive cues such as unique proteins serving as the signaling molecules that can promote the development of pancreatic tissues. It is, therefore, of paramount importance to characterize whether the instructive factors entailed in dpECM are age-associated. The protein content of ECM might also vary by sex. Another factor affecting ECM profiles is the approach adopted for decellularization. There are primarily two approaches for tissue decellularization. One utilizes a detergent-based treatment, whereas the other utilizes non-detergent methods. The detergent-based approach has been used more often to prepare dpECM [7,8,18,19,20,21,22,23,24]; however, the use of detergents might lead to the loss of many valuable proteins from the dpECM [25,26,27]. We developed a detergent-free decellularization protocol for dpECM preparation [9]. In this study, we sought to identify a better decellularization method to prepare biological samples for further interrogation of porcine dpECM proteomic profiles. We investigated gender and age differences in the porcine dpECM profile. We chose porcine pancreas for the study due to its similarities to the human anatomy and its tissue dimensions, which empower its usage as a matrix for organoid development [28]. This study offers fundamental information on pancreatic dpECM in different animal ages and genders, which will help identify the signaling molecules that are pivotal in creating tissue-specific cues for engineering tissues in vitro.

On the other hand, mass spectrometry (MS) is currently seen as the best approach for characterizing ECM composition. Mass spectrometry approaches are characterized as being either “top down”, in which intact proteins are analyzed by a mass spectrometer, or “bottom-up”, in which proteins undergo enzymatic digestion into a peptide gel for analysis. Bottom-up approaches are the most commonly used approach to quantitative proteomic analysis, as the peptide mixtures generated by enzymatic digestion fragment predictably and are easier to separate, ionize, and analyze [29,30,31]. LC-MS based approaches are by far the most common for use in proteomic analysis of tissues [32]. In this study, we used liquid chromatography-tandem mass spectrometry (LC-MS/MS), a more advanced LC-MS method, to quantify protein expression of the dpECM as opposed to other similar bottom-up approaches. This technology directly searches against large existing databases to identify protein sequences immediately, unlike non-LC–MS-based mass spectrometry methods like matrix-assisted laser desorption and ionization (MALDI) and surface-enhanced laser desorption and ionization (SELDI) [31,32]. Unlike standard LC–MS though, LC–MS/MS utilizes two mass analyzers in tandem, allows for higher flow rates, and performs further analysis by fragmentation. This also allows it to achieve higher retrieval rates of hydrophobic proteins than other bottom-up mass spectrometry approaches. In addition, LC–MS/MS is more sensitive and less labor intensive than standard LC–MS, with higher retrieval rates of hydrophobic proteins than gel-analysis based MS methods, thus making it ideal for analysis of dpECM [31,32,33].

## 2. Results

### 2.1. Comparison of Detergent-Free with Detergent-Based Decellularization of Porcine Pancreata

**Figure 1:**
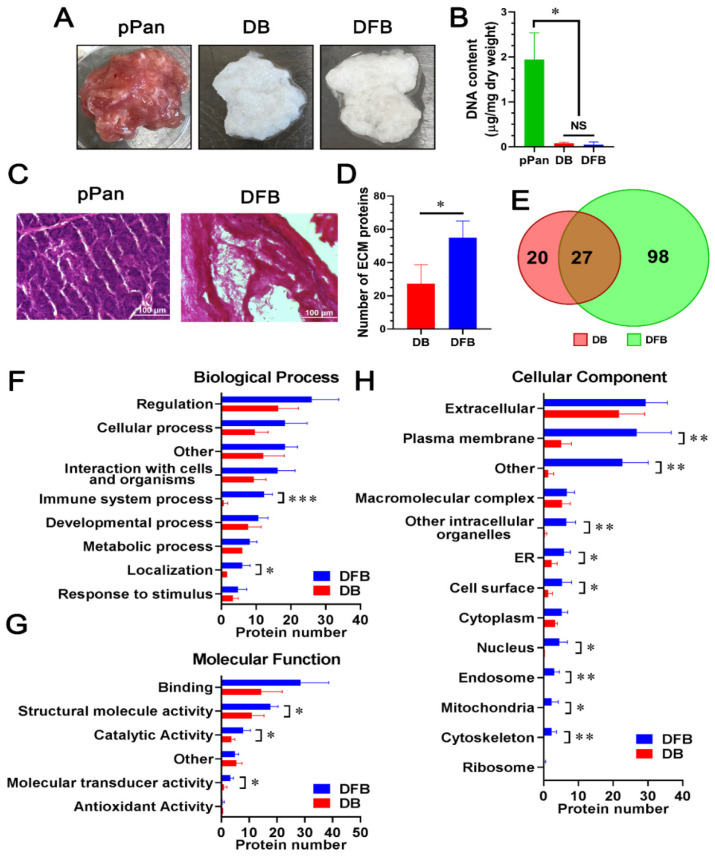
Comparison of detergent-free with detergent-based decellularization of porcine pancreata. (**A**) Porcine pancreatic tissues before (pPan) and after decellularization using a detergent-based (DB) or detergent-free method (DFB). (**B**) DNA content of pPan (*n* = 6), detergent-based (*n* = 3) or detergent-free (*n* = 6) dpECM. *: *p* < 0.05; NS: not significant. (**C**) H&E staining showing porcine pancreatic tissue morphology before (pPan) and after decellularization (DFB) with a detergent-free method. Scale bars: 100 μm. (**D**) Average numbers of extracellular proteins identified from detergent-based or detergent-free dpECM. *: *p* < 0.05. (**E**) Venn diagram showing the total numbers of extracellular proteins identified in the dpECM. (**F**–**H**) Gene ontology enrichment analysis of extracellular proteins identified in the dpECM assigned to biological process (**F**), molecular function (**G**), and cellular component (**H**). *: *p* < 0.05; **: *p* < 0.01; ***: *p* < 0.001. Data are shown as mean ± SD.

We have developed a detergent-free decellularization method that conserved more matrix proteins as compared to a detergent-based treatment [9]. This approach was used to prepare porcine pancreatic dpECM. After decellularization using either the detergent-based or detergent-free method, we observed a visual color change from the initial red/pink color of the native pancreatic tissue to completely white or light beige (Figure 1A). In order to confirm the complete removal of cells from the matrix, we determined the total DNA content in the matrix after decellularization. The total DNA content was reduced from 1937.70 ± 599.38 ng/mg in dry weight pancreas to 79.39 ± 23.04 ng/mg in decellularized ECM obtained using the detergent-based method, and 49.99 ± 58.33 ng/mg in decellularized ECM obtained using the detergent-free method (*p* < 0.001), suggesting a 95.90 ± 1.19% or 97.42 ± 3.01% DNA removal rate in the detergent-based and detergent-free decellularized ECM, respectively (Figure 1B). To confirm the removal of animal cells from the decellularized tissues, we performed hematoxylin and eosin (H&E) staining. The decellularized tissue prepared using the detergent-free method was devoid of nuclei (Figure 1C,D) as opposed to native tissue (Figure 1C pPan). The results confirmed the complete removal of animal cells from the pancreatic tissue with the detergent-free method.

Next, we performed liquid chromatography–tandem mass spectrometry (LC–MS/MS) to define the proteome of the dpECM prepared using both the detergent-based and detergent-free approaches. Some representative spectra are shown in Figure S1. We found the number of extracellular matrix proteins conserved in the detergent-free dpECM was significantly greater than those conserved in the detergent-based dpECM (*p* = 0.02) (Figure 1D, Tables S1 and S2). Among them, 27 proteins were common in dpECM prepared by both methods (Figure 1E, Tables S1 and S2). To characterize the composition of the proteins found in the dpECM, we performed gene ontology (GO) analysis using the Structural Alignment Program for Proteins (STRAP). We categorized the dpECM into biological process, molecular function, and cellular component assignments (Figure 1F–H). The GO analysis of the biological processes revealed that the number of proteins related to immune system processes (GO: 0002376) (*p* = 9.34 × 10^−5^) and localization (GO: 0051179) (*p* = 0.016) in the detergent-free group were significantly higher than those in the detergent group (Figure 1F). In the category of molecular function, more proteins were found to be involved in structural molecule activity (GO: 0005198) (*p* = 0.024), catalytic activity (GO: 0003824) (*p* = 0.038), and molecular transducer activity (GO: 0060089) (*p* = 0.029) in the detergent-free dpECM (Figure 1G). Moreover, in the category of cellular components, more proteins were enriched in the detergent-free dpECM, including but not limited to the subcategories of plasma membrane (GO: 0005886) (*p* = 0.008) and cell surface (GO: 0009986) (*p* = 0.0499) (Figure 1H). This analytic data showed that detergent-free decellularization retains more extracellular proteins, especially those involved in localization, structural molecule activity, catalytic activity, and molecular transducer activity.

### 2.2. Proteomic Analysis and Bioinformatic Characterization of dpECM Suggest That the Composition of dpECM Proteins Is Affected by Gender in Adult Tissues

**Figure 2:**
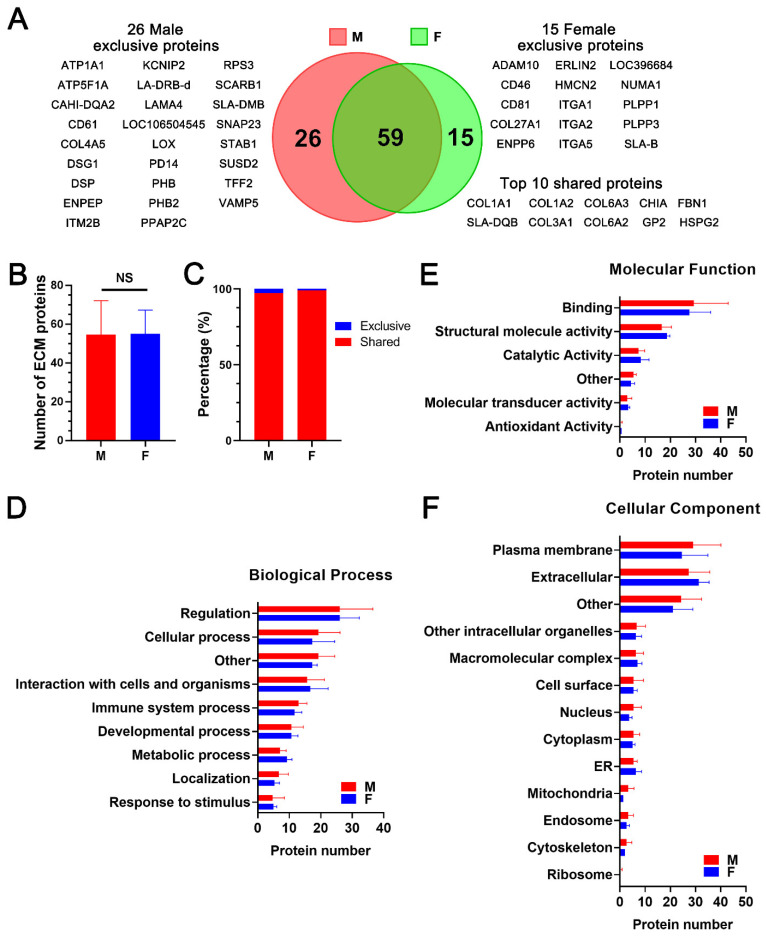
Proteomic analysis of dpECM obtained from adult male and female pigs. (**A**) Venn diagram showing total numbers of extracellular proteins identified in dpECM prepared from male or female pigs. Proteins exclusive to each gender and ten most abundant proteins shared by both genders were listed. (**B**) Average numbers of extracellular proteins identified in dpECM prepared from adult male (M, *n* = 3) and female (F, *n* = 3) pigs. NS: not significant. (**C**) Percentage of shared or exclusive proteins in the total abundance of extracellular proteins in each gender. (**D**–**F**) GO enrichment analysis of extracellular proteins identified in dpECM that are assigned to biological process (**D**), molecular function (**E**), and cellular component (**F**). Data are shown as mean ± SD.

In the next experiment, we examined whether protein composition varies between dpECM prepared from adult male and female pigs. After converting the UniProtKB entry names of the extracellular proteins into gene symbols for each sample, 85 and 74 extracellular proteins were found in dpECM obtained from male and female pigs, respectively. Among them, 59 proteins were shared by both genders (Figure 2A; Table S2). Interestingly, 26 proteins were found to be unique in the male dpECM and 15 proteins were exclusive to female dpECM. Most notable among the proteins detected only in the male animals were DSP, LOX, TFF2, ITM2B, LAMA4, PHB, and ATPA1. Drospirenone (DSP) is an anti-androgenic progestin present within males, which blocks the effects of male androgenic sex hormones [34,35]. Lysyl oxidase (LOX) is responsible for crosslinking elastin and collagen in order to form an insoluble extracellular matrix [36]. Its production has been found to be androgen dependent. An upregulation of androgens results in an upregulation of the LOX gene [37]. TFF2 hormone spreads throughout the body through the bloodstream, and has been consistently detected more in male organs than female organs [38,39]. TFF2 is known to prevent the apoptosis of insulin producing β cells and to increase β cell proliferation [40]; however, the mechanisms for β cell apoptosis, proliferation, and function are sexually dimorphic [40,41]. This may lead to a traceable expression of TFF2 in only male dpECM. ITM2B likely plays a role in male reproduction and sexual maturation [42]. Laminin a4 (LAMA4) is expressed in XY somatic cells, and thus, is associated with males [43]. Prohibitin (PHB) has been shown to play a role in the disruption of obesity related insulin homeostasis in males, but not in females [44,45]. There was a sexually based differential expression of ATPA1, which is associated with hypertension in males, but not females [46]. In contrast, ADAM10, CD46, CD81, ENPP6, PLPP1, NUMA1, ITGA2, and ITGA5 were among the proteins detected solely in the female animals. ADAM10 was detected in all female dpECM samples. Previous studies have shown that ADAM10 is sensitive to estrogens, but not androgens [47,48]. Androgen levels do not alter ADAM10 expression, while ovariectomies in female mice resulted in an increase in ADAM10 mRNA transcription [47,48]. The CD46 and CD81 we found to be expressed in pancreas play a role in fertilization [49,50,51]. Interestingly, ENPP6, an enzyme associated with the ovaries was detected in female dpECM groups [52]. Other studies have reported that PLPP1, NUMA1, ITGA2 and ITGA5 are expressed at high levels in female animals [53,54,55,56,57], which may have made them detectable in the female dpECM groups.

Additionally, based on the relative abundance of each protein, the top 10 proteins commonly existing within dpECM in both genders are collagens (including COL1A1, COL1A2, COL3A1, COL6A2, and COL6A3), fibrillin-1 (FBN1), pancreatic secretory granule membrane major glycoprotein proteins (GP2), heparan sulfate proteoglycan 2 (HSPG2), chitinase acidic (CHIA), and MHC class II antigen swine leukocyte antigen (SLA-DQB) (Figure 2A). There was no significant variation in the total number of ECM proteins in male and female dpECM based on the ECM category of the gene ontology (GO) (GO: 5576) (Figure 2B and Table S2). Approximately 97% of proteins in the ECM category (GO: 5576) were commonly present in the two gender groups (Figure 2C). Furthermore, GO enrichment analysis unveiled that there was no significant difference in total protein number within the categories of biological process, molecular function, or cellular component assignment (*p* > 0.05) (Figure 2D–F). These results suggest that gender affects the overall proteomic composition of dpECM, but ECM proteins are similar in males and females.

### 2.3. Comparison of dpECM Proteins Detected inYoung and Adult Porcine Pancreata

**Figure 3:**
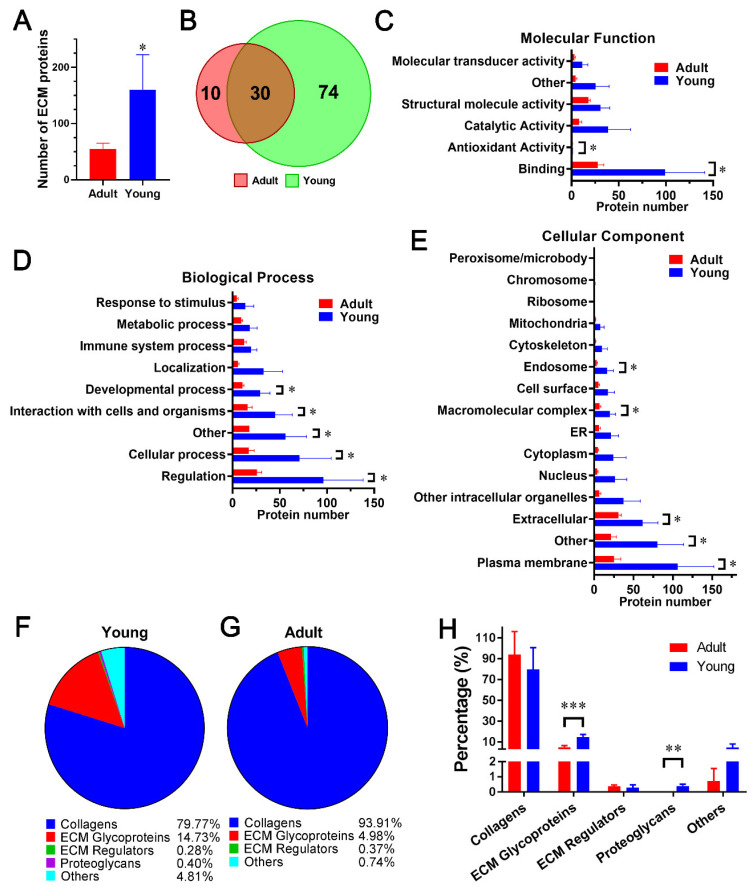
Comparison of dpECM proteins detected in young and adult porcine pancreata. (**A**) Average number of proteins identified in adult (*n* = 4) and young (*n* = 4) dpECM. *: *p* < 0.05. (**B**) Venn diagram showing total numbers of extracellular proteins commonly identified in the adult and young dpECM. (**C**–**E**) GO enrichment analysis of proteins identified in the dpECM assigned to molecular function (**C**), biological process (**D**), and cellular component (**E**). *: *p* < 0.05. (**F**,**G**) Proteins in young (**F**) and adult (**G**) dpECM involved in the developmental process were classified into indicated matrisome subcategories. (**H**) Percentage of protein abundance in the total abundance of dpECM proteins involved in the developmental process. **: *p* < 0.01; ***: *p* < 0.001. Data are shown as mean ± SD.

In the next experiment, we interrogated whether the composition of dpECM is age dependent. We prepared pancreatic dpECM from adult (*n* = 4) and young (*n* = 4) pigs. We detected more than 160 ± 62 proteins in young dpECM, whereas only 55 ± 10 proteins were identified in adult dpECM (Figure 3A, Tables S2 and S3). Any protein that was detected in at least three out of four animals in the young or adult cohorts was considered a common protein. There were 104 and 40 common proteins found in the young (*n* = 4) and adult (*n* = 4) dpECM, respectively. Of those, 30 were shared between both cohorts (Figure 3B, Tables S2 and S3). These results suggested that young porcine pancreatic tissue contains more extracellular proteins than adult dpECM. GO enrichment analysis revealed the dpECM protein distribution in the categories of molecular function, biological process, and cellular component (Figure 3C–E and Table S4). In the category of molecular function, more proteins involved in antioxidant activity (GO: 0016209) (*p* = 0.0498) and binding (GO: 0005488) (*p* = 0.041) were present in young dpECM than in adult dpECM (Figure 3C). Likewise, in the category of biological process, proteins related to cellular process (GO: 0009987) (*p* = 0.048), developmental process (GO: 0032502) (*p* = 0.032), interaction with cells and organisms (*p* = 0.021), regulation (GO: 0065007) (*p* = 0.044), and other (*p* = 0.041) were more present in the dpECM of young pigs than in adult dpECM (Figure 3D). By analyzing cell activity-associated proteins, we found that more proteins in the young dpECM were associated with the endosome (GO: 0005768) (*p* = 0.041), plasma membrane (GO: 0005886) (*p* = 0.037), extracellular region (*p* = 0.0491), and macromolecular complex (*p* = 0.032) compared to the adult dpECM (Figure 3E). This data demonstrated that the composition of the dpECM is age-dependent, and that young dpECM contains unique proteins associated with specific cellular activities or biological processes for tissue or organ development.

We further characterized the proteins in the subcategory of developmental processes using MatrisomeDB 2.0. We identified 22 developmental process proteins from young dpECM and 14 from adult dpECM. Collagens have the highest proportion of dpECM proteins. They have a relative abundance of 79.77% in young and 93.91% in adult dpECM, respectively (Figure 3F–H). ECM glycoproteins had a relative abundance of 14.73% in young and 4.81% in adult dpECM, respectively (Figure 3F,G). Thus, the proportion of ECM glycoproteins (*p* = 0.0005) in the young dpECM was significantly higher than that in the adult dpECM. Proteoglycans were undetectable in the adult dpECM (Figure 3H). Overall, collagens were markedly enriched as extracellular proteins associated with developmental processes in both the young and adult dpECM, implying that collagens play a vital role in the pancreatic tissue microenvironment.

**Figure 4:**
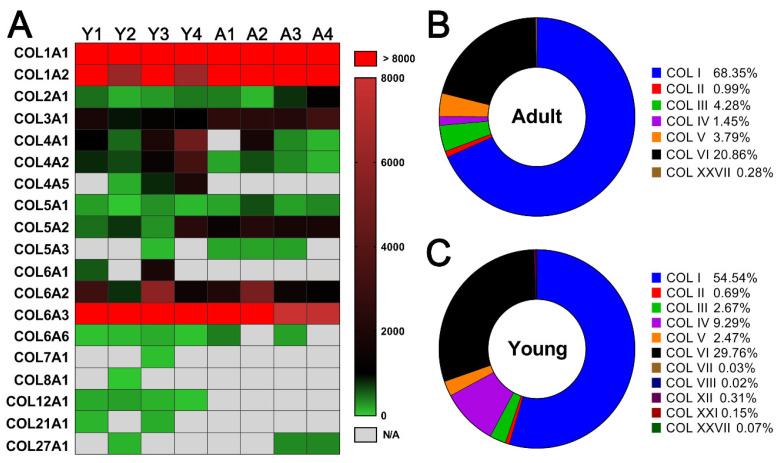
Comparison of collagen contents in young and adult dpECM. (**A**) Heatmap of collagens identified in young (Y1–Y4) (*n* = 4) and adult (A1–A4) (*n* = 4) dpECM. Columns represent individual dpECM. The relative abundance of each molecule is depicted by a color code. Undetected collagens are shown in grey. (**B**,**C**) Average percentage of individual collagen type in the total abundance of collagens in adult (**B**) and young (**C**) dpECM.

Accordingly, we interrogated the relative abundance of all types of collagens detected in young and adult dpECM. As shown in Figure 4A, collagens I–VI were the most common collagens in both young and adult dpECM. Collagen XII (all four samples) and XXI (two out of four samples) were detected only in the young dpECM, whereas collagen XXVII was detected in the adult dpECM (two out of four samples) with a higher abundance than young dpECM. Type I–VI collagens made up 99.43% and 99.72% of total collagen content in young and adult dpECM, respectively (Figure 4B,C). These results demonstrated that type I through VI collagens are dominant among all the collagens in both young and adult dpECM.

### 2.4. Enriched Pathway Analysis for Young and Adult dpECM

**Figure 5:**
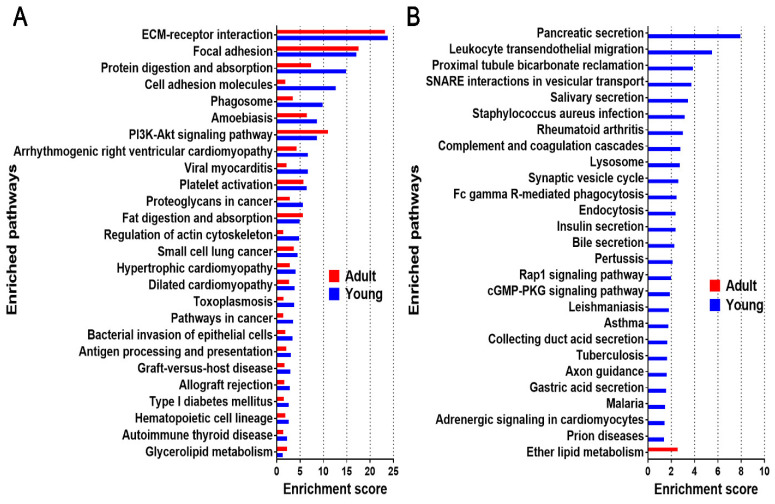
Enrichened pathway analysis in young (*n* = 4) and adult (*n* = 4) dpECM. (**A**) The enriched pathways identified in both young and adult dpECM. (**B**) The enriched pathways identified solely in young or adult dpECM. The bars show enrichment scores calculated by [−log10 (*p*-value)] for individual pathways. These enriched KEGG pathways were confirmed using the Modified Fisher Exact test (EASE score *p* < 0.05).

A microenvironment that is constituted by ECM has an intimate relationship with various cellular signaling pathways [4,58,59]. To understand the signaling pathways regulated by these bioactive materials, we performed modified Fisher Exact tests using the Database for Annotation, Visualization, and Integrated Discovery (DAVID) to measure the molecular enrichment in each annotation term and the modified Fisher Exact *p*-value for each pathway. Enrichment scores (−log10 (*p*-value)) of the enriched pathways (*p* < 0.05) in young and adult dpECM are shown in Figure 5A. We found 26 pathways enriched in both the young and adult dpECM. Among them, the enrichment scores of both the cell adhesion molecules (CAMs) and the regulation of actin cytoskeleton demonstrated that these two pathways had the greatest differences in young and adult dpECM. There were 26 unique enriched pathways found in the young dpECM, including pancreatic secretion, leukocyte transendothelial migration, proximal tubule bicarbonate reclamation, SNARE interactions in vesicular transport, and insulin secretion, among others (Figure 5B). In contrast, only the ether lipid metabolism pathway was found to be unique to adult dpECM (Figure 5B). 

We next chose two pathways designated as pancreatic secretion and insulin secretion pathways for further analysis using the KEGG analytic software. Molecules presented in both the young and adult dpECM were given in magenta boxes, and proteins presented only in young dpECM were marked in blue boxes (Figure S2). This indicated that young not adult dpECM proteins were involved in the pancreatic and insulin secretion pathways. In the pancreatic secretion pathway, 12 proteins, including ATP, PMCA (plasma membrane Ca^2+^-ATPases), CD38/157, IP_3_R, Rap1, CPA, CPB, PNLIP, PLRP1, PLRP2, PLA2, and NBC1 (Figure S2A), were recognized in the young dpECM. PMCA plays an important role in regulating Ca^2+^ concentration in cells [60], whereas IP_3_R is an inositol 1,4,5-trisphosphate (IP_3_) receptor [61]. ATP, PMCA, and IP_3_R are crucially associated with calcium signaling and pancreatic functions [61]. RAP1 is known to be localized in pancreatic zymogen granules and is required for pancreatic amylase release [62]. Carboxypeptidase A (CPA), carboxypeptidase B (CPB), and PNLIP are the pancreatic proteases and lipases that are produced in the pancreatic secretion pathway. Enrichment of these proteins in young but not adult dpECM suggests age-specific roles in regulating calcium ion concentration and enzymes that are critical for pancreatic functions.

In the insulin secretion pathway, there were six proteins unique to young dpECM: ATPase, GLUT1/2, glucagon-like peptide–1 (GLP-1), Gq/G11, IP_3_R, and Syntaxin (Figure S2B). GLUT1/2 mediates glucose transport, leading to dynamic changes of ATP concentration inside β cells and stimulating insulin secretion [63]. GLP-1 is a peptide that can greatly improve β cell function [64]. Gq/G11 has been found to be essential for glucose responsive insulin secretion in mice [65]. Syntaxin participates in the priming and fusion events for insulin release, a tertiary SNARE complex formed by Syntaxin, SNAP25, and vesicle-associated membrane protein (VAMP2), known to be vital to glucose-stimulated insulin secretion from β cells [66]. Taken together, these data suggest that the young dpECM retained magnified extracellular stimuli for islet cell maturation.

**Figure 6:**
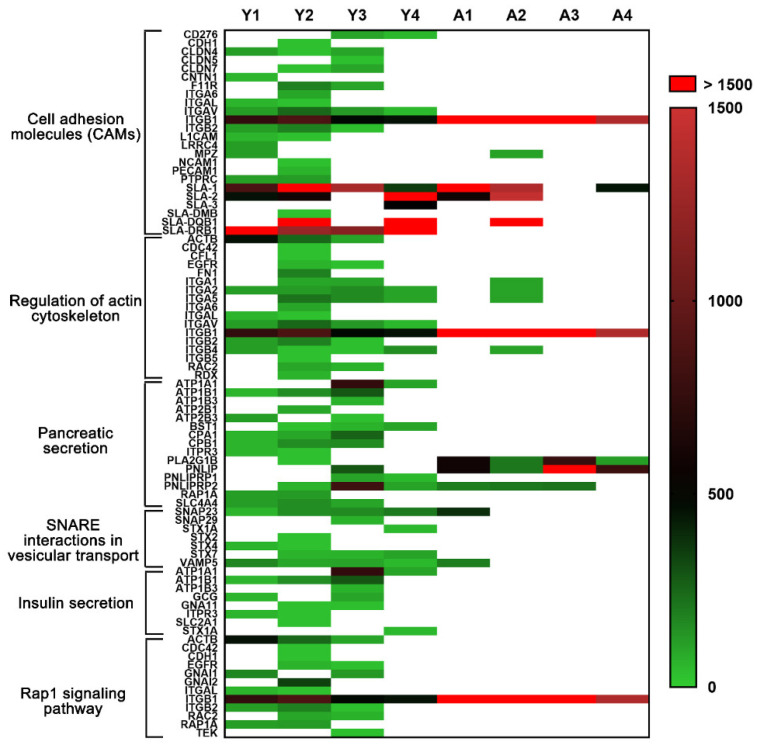
Heatmap of extracellular proteins in six enriched pathways identified in young (*n* = 4) or adult (*n* = 4) dpECM. Columns represent different dpECM samples from young (Y) or adult (A) pigs, whereas rows represent different gene symbols of ECM proteins. Several proteins were found in more than one pathway. The relative abundance of each protein is depicted by the heatmap color scale. White color stands for the proteins not detected in the samples.

As displayed in Figure 5B, the pathways pertaining to pancreatic secretion, SNARE interactions in vesicular transport, insulin secretion, and Rap1 signaling were uniquely enriched in the young dpECM. These pathways play important roles in regulating pancreatic cell–cell interactions as well as exocrine and endocrine functions. Hence, we categorized the proteins associated with these pathways in young and adult dpECM (Figure 6). CAMs are important mediators of cell–cell interactions. They determine the cell fate by regulating growth, differentiation, and organization within tissues [67]. The structure of the actin cytoskeleton in a cell is highly dependent upon various signals and cellular processes, such as insulin exocytosis [68]. By comparing the relative abundance of all the proteins involved in these six pathways, we found that 14 of these proteins were in the adult dpECM, whereas 64 were detected in the young dpECM (Figure 6). All the proteins present in adult dpECM were correspondingly present in young dpECM. Among these proteins, various types of integrins were detected in the young dpECM, but not in the adult ones (Figure 6). They include integrin subunits α1/2/5/L/V (ITGA1, ITGA2, ITGA5, ITGAL, and ITGAV); and integrin subunits β1/2/4 (ITGB1, ITGB2, and ITGB4). By contrast, the adult dpECM contained only ITGB1. In addition, we observed that synaptosome associated protein 23 (SNAP23) as well as Syntaxin 4 and 7 (STX4 and STX7) were present in the young dpECM (Figure 6). Exocytosis mediated by SNARE is essential for hormone and enzyme secretion from cells [69]. Our data suggested that young dpECM contains relatively more proteins that regulate islet cell functionalities. 

### 2.5. Potential Protein Networks Enriched in Young and Adult dpECM

**Figure 7:**
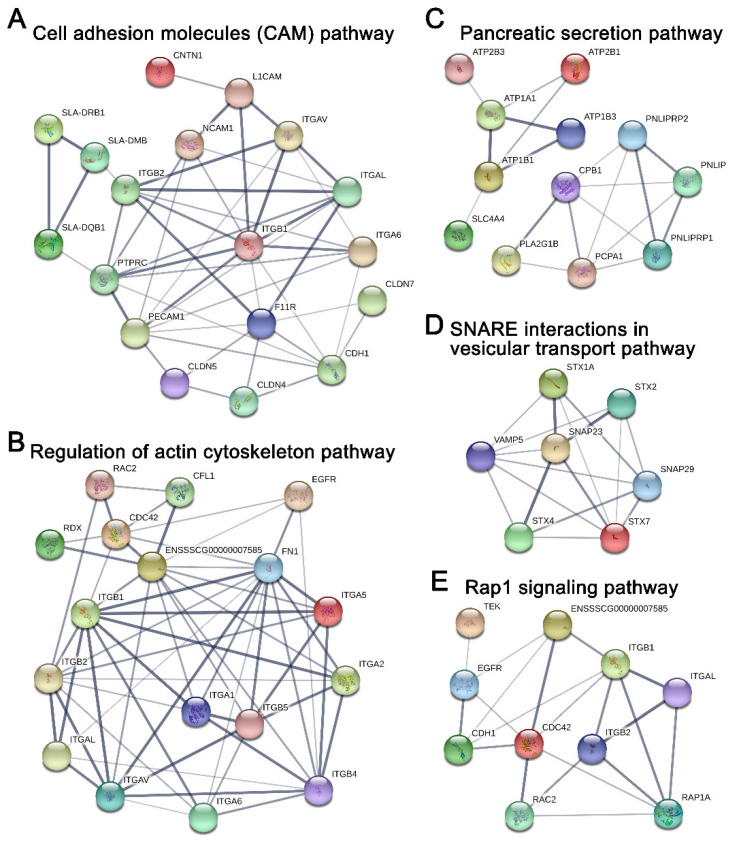
Association networks of the proteins in the enriched pathways identified from young dpECM proteins detected. (**A**) Cell adhesion molecule pathway. (**B**) Regulation of actin cytoskeleton pathway. (**C**) Pancreatic secretion pathway. (**D**) SNARE interactions in vesicular transport pathway. (**E**) Rap1 signaling pathway. The altered colors stand for different proteins. Line thickness represents the degree of supporting data from publications. Due to the difference in the annotations of protein identities in the DAVID and STRING databases, PCPA1 in (**B**) refers to carboxypeptidase A1 (CPA1); ENSSSCG00000007585 in (**B**,**E**) refers to actin β (ACTB).

To ascertain the potential association network of proteins involved in the aforementioned pathways in young dpECM, we characterized the protein–protein interactions and their involvement in selected pathways using STRING (Figure 7). All of the proteins shown in the protein interaction networks were identified from the dpECM samples. Cell adhesion molecules play important roles in a variety of biological processes. They support tissue structure and function, and deliver signals to coordinate varied cell activities. As revealed by Figure 7A, many cell adhesion molecules, including receptors such as ITGAV, ITGAL, ITGA6, ITGB1, ITGB2, CDH1, CLDN4, and NCAM1, etc., were enriched in young dpECM. Further analysis on the actin cytoskeleton pathway revealed that numerous integrin receptors, including ITGA1, ITGA2, ITGA5, ITGA6, ITGAL, ITGAL, ITGB1, ITGB2, ITGB4, and ITGB5 identified in the young dpECM are involved in actin cytoskeleton signaling (Figure 7B). Our analysis suggested that CDC42, RAC2, and EGFR may also play a role in regulating the actin cytoskeleton pathway (Figure 7B). Furthermore, we found that a number of sodium/potassium-transporting ATPases (ATP1A1, ATP1B1, and ATP1B3) and calcium-transporting ATPases (ATP2B1 and ATP2B2) were enriched in the young dpECM. In addition, protein CPB1, which was reported to be able to facilitate pancreatic cell survival and proliferation, was found in the pancreatic secretion pathway and detected in the young dpECM [70] (Figure 7C). It appears that several dpECM proteins were involved in SNARE interactions in the vesicular transport pathway (Figure 7D). SNARE23 supports the fusion of insulin secreting granules with the plasma membrane for insulin exocytosis in pancreatic β cells [71]. Proteins involved in granule fusion to the plasma membrane, such as VAMP5 [72], STX1A, STX2, and STX4 [73], were found in this vesicular transport signaling pathway (Figure 7D). They are associated with calcium-dependent hormone secretion. Furthermore, we examined dpECM proteins’ involvement in the Ras-associated protein-1 (Rap1) signaling pathway, as Rap1 plays a central role in the control of cell–cell and cell-matrix interactions which regulate receptors’ signaling (Figure 7E). The protein network analysis unveiled the association of integrins such as ITGB1, ITGB2 and ITGAL, and receptors such as CDH1 and EGFR along with small GTPases CDC42 and Rac2 detected in the young dpECM within the Rap1 signaling pathway (Figure 7E). These analyses indicated that many proteins present in the young dpECM were involved in cellular signaling pathways and were intimately interrelated in these pathways (Figure 7). In addition, the evaluation of protein–protein association in the six pathways shown in Figure 7 unveiled age-dependent differences in key signaling proteins. Approximately 61 proteins recognized in young dpECM were found in the protein–protein interaction network (data not shown). Moreover, proteins in the CAM, regulation of actin cytoskeleton, and Rap1 signaling pathways were closely interrelated according to STRING analysis. ITGB1, ITGB2, and ITGAL were involved in the three pathways. In addition, ATP1A1, STX1A, SNAP23, ITGAV, and CDH1 were also involved in these signaling networks (data not shown). In contrast, only eight proteins in the adult dpECM were associated with these networks. These observations confirmed our bioinformatic analyses shown in Figure 5, Figure 6 and Figure 7 and demonstrated the superior bioactivity of young dpECM as compared to adult dpECM in regard to enriched proteins involved in a variety of signaling pathway networks.

## 3. Discussion

In this study, we demonstrated the advantage of the non-detergent treatment to preserve more porcine tissue matrix proteins during decellularization. This is noteworthy, as many important proteins would be overlooked if they were not retained during decellularization. Detergent-based decellularization is a conventional method for preparing native biomaterials [5,18,74,75]; however, detergents, particularly SDS, may disrupt the cells and alter the conformation of the proteins that bind to cells and/or ECM. Transmembrane proteins including receptors like integrins would be removed due to the use of detergent. Growing evidence suggests that detergent is harsh on the preservation of proteins such as growth factors and cytokines in dpECM [9,25,26,76]. Hence, we developed an osmotic pressure-based method, which ruptures cells through repeated treatment using a high osmotic pressure solution (10% NaCl and 0.1% NH_4_OH) and deionized water for the decellularization of ECM [9]. We found that many proteins located in the plasma membrane, on the cell surface, and in the cytoskeleton were preserved during decellularization when treated with this detergent-free method, allowing proteomic analysis of proteins involved in cell-ECM interactions. 

Interestingly, a few proteins in dpECM were found to be associated with the nucleus, endosome, and other intracellular components as shown in Figure 1H. There are two potential reasons for these observations. First, the compartmentalization of a protein within a cell is a dynamic process. A protein can be localized in the nucleus or cytoplasm or be transported to an extracellular space at any given time, depending upon the biological events that the protein is involved in and the needs of the cell to function and survive. For example, synaptosomal-associated protein is the one detected in dpECM. It is annotated as a protein in both the nucleus and the plasma membrane by the STRAP program. Another example is Caveolin-1. It belongs to both the plasma membrane and Golgi apparatus according to the STRAP database. Therefore, it is possible that an intracellular or nuclear protein can also be found in the extracellular matrix. Second, the decellularization procedure could also affect the protein enrichment of decellularized ECM. For instance, it was found that a relative abundance of ECM proteins in Zebrafish heart was altered after being treated with a detergent for decellularization. Both cytoplasmic and nuclear proteins’ relative abundance decreased in these detergent-treated ECMs [77]. Our study on Matrigel proteomics also suggested the enrichment of intracellular proteins, including those inside the nucleus and chromosomes in Matrigel, a well characterized ECM matrix prepared from mouse basement membrane [10].

Decellularized pancreatic extracellular matrix has been widely used for tissue regeneration. Nevertheless, the impacts of gender and age on the protein content of dpECM have remained largely elusive. It has long been recognized that organ development is associated with age [78,79,80]. Thus, the ECM is adapted for organ development and function at different ages. The influence of sex on dpECM is another factor that has not been fully investigated. In this study, we systematically interrogated the influences of gender and age on dpECM through proteomic and bioinformatic analysis. We discovered that the young dpECM contains approximately 68.5% more extracellular proteins than adult dpECM, implying these proteins may play a role during pancreatic organ development in young animals. Our study results also indicated that the dpECM protein profile is sex-dependent. 

Of all collagens that make up the ECM, fibrillar collagens (I-III, V) and basement membrane collagen (IV) are considered the most important. While other pancreatic proteome analyses suggested that several detergent-based decellularization techniques remove basement membrane and matricellular proteins, particularly collagen VI [20], our analysis identified each of these collagens in abundance within our decellularized samples, further confirming a successful decellularization with limited removal of the critical ECM components. Collagens I–IV were dominant among all detected collagens. This is similar to results shown by other studies [20,81,82,83]. In total, our decellularization yielded 19 different subtypes of collagen. This is an improvement over other attempts to quantify the protein sequence coverage of collagen in dpECM, which have, to our knowledge, produced fewer subtypes of collagen [20,81,82,83]. As the pancreatic proteome spans several orders of magnitude, it is expected that the proteomic sequence coverage will vary not only between samples within each study, but especially between different studies. While discrepancies in which subtype collagens exist between all studies, unlike any known previous study, our decellularization yielded COL6A6 and COL27A1. As mentioned previously, we have reported that collagen V is critical for the generation of biologically functional human islet organoids from iPSCs and hESCs [10]. Our decellularization produced collagen V content either on par with or superior to other decellularization methods. The relative abundance of collagen content in adult pancreases of human, pig, and mouse have been found to be above 90%, which is in accordance with our findings [10,20,81,82,83]. This further exemplifies the importance of the different relative abundances of collagen found by our analysis. 

KEGG pathway analysis suggested that pathways such as the cell adhesion molecule and pancreatic secretion pathways found in both young and adult groups are enriched 5-fold, along with 24 unique pathways that are enriched in young dpECM. This finding is consistent with very recent studies analyzing cardiomyocyte [84], kidney [85], pancreas [82], artery [86], and meniscus [87] decellularized ECM prepared from different ages. Li et al. investigated the difference between ECM components in human pancreases among four age groups: fetal (18–20 weeks gestation), juvenile (5–16 years old), young adult (21–29 years old) and older adult (50–61 years old). They discovered that 84 ECM proteins were significantly altered throughout the fetal, juvenile, and adult stages [82]. Interestingly, we found that some proteins associated with the insulin secretion pathway were enriched in young dpECM. This implies that dpECM in young animals supports pancreatic development, as insulin is one of the key enhancers for stem cell proliferation and differentiation [88,89,90]. Additionally, we observed the enrichment of CAMs and many proteins involved in pancreatic secretion pathways in young dpECM. We speculated that integrins and Syntaxin found from young dpECM might be critical to these enrichments based on protein–protein interaction network analyses. In addition, we showed the presence of ITGB1, STX1A, and SNAP23 in young dpECM. Integrin ITGB1 provides physical support for cell adhesion and regulates cell proliferation and development by transmitting signals bidirectionally between the ECM and intracellular environment [91,92,93]. ITGB1 was found to be essential to islet development, integrity, and function in both rodent and human [91,94]. STX1A is a part of the VAMP2–STX1A–SNAP25 SNARE complex. It is vital to insulin release, as it mediates the docking of insulin secretory granules and hormone exocytosis. [95] Moreover, SNAP23 plays an important role in facilitating the fusion of granules to the plasma membrane during insulin exocytosis. The loss of SNAP23 in the exocrine pancreas suppresses the fusion of granules to the plasma membrane [69]. Hence, ITGB1, STX1A, and SNAP23 are important to pancreatic development and function. These could be good targets for elevating maturity and improving the function of islets developed from iPSCs. In addition, a line of studies suggested the ubiquitous presence of type I-V collagens in human, porcine, and rat pancreatic ECM [6,9,81], hinting at their potential roles in regulating pancreas development and functions. We reported that based on proteomics and stem cell biology, type V collagen has been identified as a critical regulator for the generation of biologically functional human islet organoids from iPSCs and hESCs [10]. 

In the attempt to understand the mechanisms of age-related effects on dpECM, it has been reported that ECM aging results in collagen deposition, increases crosslinking, and increases collagen degradation as a result of post-transcriptional modifications [96,97]. Although not yet fully understood, several molecular mechanisms for this behavior have been investigated. The most well studied mechanism by which age impacts ECM composition stems from the production of free radical reactive oxygen species (ROS). ROS is produced during normal cellular function at all stages of life and is highly reactive due to its unpaired electrons. With increasing age, the secretion of antioxidant enzymes decreases, and exposure to external agents which amplify ROS production such as infection, smoke exposure, and chemical exposure increases [96,97]. As a result of its reactivity and instability, ROS is responsible for most ECM protein oxidation and degradation. ROS alters the regulation of the matrix metalloproteinases (MMPs) which degrade ECM components and thus reduces its biomechanical properties [98]. Increased exposure to ROS may explain why this study found a lower total collagen content in the adult dpECM than in young dpECM, as shown in Figure 3F,G and Figure 4B,C. 

The mechanism for sexual impact at the ECM level has not been thoroughly investigated, but sex has been found to influence ROS production in tandem with age. The reduction in ovarian hormone production following menopause causes downstream metabolic disturbances in human females. This leads to a further increase of free radicals that produce ROS and induct MMPs. Lower expression of female sex hormones results in a decrease in type I and III collagens in particular [14].

It is worth noting that the number and type of ECM proteins detected by a mass spectrometer are dependent on both the instruments and proteomics approaches applied [9]. With the improvement in mass-spectrometric instrumentation, some trace ECM proteins that are undetectable using a conventional mass spectrometer can be identified by a more advanced mass spectrometer. 

Taken together, this study revealed that the extracellular matrix protein content of porcine pancreas varies significantly depending not only on age but also gender. Young dpECM contains more proteins than adult dpECM. These proteins are directly associated with various signaling pathways responsible for regulating pancreatic functionality. In addition, detergent-free decellularization allows for preserving more bioactive proteins in ECM. Moreover, enrichment pathway analysis unveiled the involvement of multiple signaling molecules, such as integrins, in a variety of biological processes. This study provides pivotal bioinformatics of porcine pancreatic microenvironments. More comprehensive characterizations are required to identify new signaling molecules that are indispensable for creating tissue-specific cues for in vitro generation of human tissues. For increased accuracy of the proteomics evaluation, quantitative analysis by mass spectrometry will be performed to measure the protein abundance and further characterize the biological samples.

## 4. Materials and Methods

### 4.1. dpECM Preparation 

Porcine pancreata were acquired from the Midwest Research Swine LLC (Gibbon, MN USA). They were collected from adult (7 months old) and young (7 weeks old) male and female pigs. All pancreata were stored at −80 °C until use. The frozen pancreata were cut into pieces under sterile conditions at 4 °C, as described in our previous work [10]. For the detergent-based treatment, the tissues were rinsed with 0.5% (*w*/*v*) sodium dodecyl sulfate (SDS) solution 5 times for 1 h each, followed by a wash with 1% (*v*/*v*) Triton X-100 solution once for 2 h. For the detergent-free decellularization, the tissues went through 4 cycles of hyper/hypotonic washes, consisting of a rinse with 10% sodium chloride (VWR International, Radnor, PA, USA) containing 0.1% ammonium hydroxide (Fisher Scientific, Pittsburgh, PA, USA) for 12 h and then with deionized water for another 12 h. The tissue samples from both methods were further washed with deionized water 10 times to completely remove any residue detergent or hypertonic solution. The dpECM was prepared by lyophilizing the decellularized tissues using a freeze dryer (LABCONCO, Kansas City, MO, USA), then grinding the tissues into powder using a Wiley Mini Mill (Thomas Scientific, Swedesboro, NJ, USA). It was stored at −20 °C until use.

### 4.2. DNA Quantification and Eosin Staining

To evaluate the efficiency of decellularization, DNA was extracted from lyophilized dpECM or untreated tissues using a QIAamp DNA Mini Kit (QIAGEN, Venlo, Netherlands). The total DNA content was measured using a Synergy H1 Microplate Reader (BioTek, Winooski, VT, USA). To perform the Hematoxylin and Eosin (H&E) staining, biological samples were fixed in 4% paraformaldehyde overnight, followed by an overnight incubation in a 30% sucrose solution (*w*/*v*). Optimal Cutting Temperature (OCT) compound (Thermo Fisher Scientific, Waltham, MA, USA) was added to the samples and stored at 4 °C overnight. This was followed by snap freezing in liquid nitrogen. The resultant frozen samples were cut into 7 μm sections on TruBond TM (Electron Microscopy Sciences, Hatfield, PA, USA) and H&E staining were conducted to confirm the removal of animal cells. 

### 4.3. dpECM Reconstitution 

Pepsin (Sigma-Aldrich, St. Louis, MO, USA, catalogue number P6887) was solubilized in 0.02 N acetic acid at 2 mg/mL. To dissolve the dpECM powder, 40 mg dpECM powder was digested in 2 mL of 2 mg/mL pepsin solution and stirred at 25 °C for 48 h, as described elsewhere [10,99]. The resultant dpECM solution was aliquoted and stored at −80 °C. The total protein content in the reconstituted dpECM was determined using a Pierce BCA protein assay kit (ThermoFisher Scientific, Waltham, MA, USA).

### 4.4. Sample Preparation for Mass Spectrometry

The dpECM (100 µg) was lyophilized to 30 µL then denatured with 20 µL of 1% SDS at 50 °C for 15 min. Disulfide bonds were reduced by adding 5 µL of 200 mM dithiothreitol to the solution, followed by incubation at 55 °C for 1 h. Cysteine thiol groups were alkylated by adding 5 µL of 375 mM iodoacetamide, followed by incubation at 40 °C for 1 h. Reduced and alkylated proteins were then precipitated by adding 370 µL of chilled acetone to the solution, followed by centrifugation at 15,000 rpm for 30 min. The resultant pellets were kept at −20 °C overnight, then centrifuged again. The supernatant was discarded to obtain protein pellets. A portion of 100 µL of 50 mM ammonium bicarbonate was added to the protein pellets and vortexed before digestion. Proteolysis was performed by adding 10 µL of 0.05 µg/µL Pierce Trypsin Protease (ThermoFisher Scientific) (with a 1:1 ratio of enzyme:protein) in two steps: first a 4 h incubation, followed by a second addition of trypsin for an additional 16 h at 37 °C using a shaker at 450 rpm. The resultant solution was vacuum dried, followed by reconstitution in 300 µL of 0.1% trifluoroacetic acid, and then fractionated into 8 fractions using a Pierce High pH Reversed-Phase Peptide Fractionation kit following the manufacturer’s protocol (Thermo Fisher Scientific). The flow-through, wash solution, and collected factions were vacuum dried and stored at −20 °C until use.

### 4.5. Liquid Chromatography–Tandem Mass Spectrometry (LC–MS/MS)

The peptide analysis was carried out using an Ultimate 3000 RSLCnano system (Dionex/ThermoFisher Scientific) coupled with a Q Exactive HF mass spectrometer (Thermo Fisher Scientific). The MS data was collected on the Q Exactive HF mass spectrometer using data-dependent acquisition as follows. Four µg peptide mixtures were dissolved in 20 µL of 0.1% (*v*/*v*) formic acid (FA). Each sample fraction was reconstituted in the peptide-containing FA solution. An aliquot of 10 µL was used for nanoLC–MS/MS analysis. The samples were loaded on an Acclaim PepMap trap column and washed with water containing 0.1% FA and 2% acetonitrile (ACN) (*v*/*v*/*v*) at 3.5 µL/min for 7 min. The peptide separation was performed using a nano-analytical column, the EASY-Spray Acclaim PepMap RSLC, by applying a gradient consisting of solvents A (HPLC-grade water with 0.1% FA) and B (HPLC-grade ACN (80%) with 0.1% FA) from 2–40% solvent B at 300 nL/min over 127 min, followed by a 3 min linear increase of ACN. For ionization, an EASY-Spray ES233 (Thermo Fisher Scientific) was used with a voltage of 2 kV and a capillary temperature of 350 °C. Full MS scans were acquired in an Orbitrap mass analyzer over an *m*/*z* range of 375–1800 with a resolution of 60,000 at *m*/*z* 200. The target automatic gain control value of 3 × 10^6^ was used with a maximum allowed injection time (Maximum IT) of 100 ms. For MS/MS, an isolation window of 1.2 *m*/*z* was utilized. The twenty most intense peaks (TopN) with a charge state between 2 and 6 were selected for fragmentation in the higher-energy collisional induced dissociation cell with a normalized collision energy of 27. The tandem mass spectra were acquired with a fixed first mass of *m*/*z* 150 in the Orbitrap mass analyzer with a resolution of 30,000 at *m*/*z* 200 and an automatic gain control of 2 × 10^5^. The ion intensity selection threshold was 2 × 10^4^, and the maximum injection time was 50 ms. All the systems were fully controlled by Thermo Xcalibur 4.0 (ThermoFisher Scientific).

### 4.6. Proteomic Data Analysis

The data were processed with a specific workflow designed in Proteome Discoverer 2.2 (Thermo Fisher Scientific). MS/MS data was interpreted using the Sequest HT search engine (Thermo Fisher Scientific) with precursor and fragment mass tolerances set at 10 ppm and 0.05 Da, respectively. A semi-trypsin peptide search was performed in Sus scrofa (taxonomy ID: 9823) protein sequence databases downloaded from UniProt (https://www.uniprot.org/, accessed on 8 May 2019) and from the National Center for Biotechnology Information (NCBI) (https://www.ncbi.nlm.nih.gov/, accessed on 18 May 2019). The following dynamic modifications of peptides were searched: carbamidomethylation on cysteine, acetylation on protein N-terminal, and oxidation on methionine. The target–decoy database search allowed us to control and estimate the false discovery rate (FDR) at 1% for peptides and proteins. Reagents used in sample preparation such as pepsin, trypsin, and keratins were excluded from the data analysis [19]. Proteins were considered identified when at least one unique peptide was present in the samples. We calculated the relative protein abundance according to a previous study [10], as a percentage derived from the spectral count of each identified protein over the total number of spectra in the entire sample. The NCBI accession numbers of obtained proteins were converted into UniProtKB entry names for gene ontology (GO) enrichment analysis, which was performed using STRAP 1.5 [100], and protein GO annotations were extracted from UniProt using the Mass Spectrometry Data Analysis (MSDA) tools website https://msda.unistra.fr (accessed on 28 May 2019) [101]. Proteins annotated with gene ontology terms GO: 5576 (extracellular region), GO: 5615 (extracellular space), GO: 5581 (collagen trimer), GO: 5886 (plasma membrane), and GO: 9986 (cell surface) that are classified as extracellular proteins were used for quantification and further analysis. Matrisome annotations were performed using MatrisomeDB 2.0 (http://www.matrisomedb.org, accessed on 2 June 2019) [10,102]. Kyoto Encyclopedia of Genes and Genomes (KEGG) pathway analysis was performed using DAVID 6.8 (Database for Annotation, Visualization, and Integrated Discovery; https://david.ncifcrf.gov/home.jsp, accessed on 26 January 2020) [103]. A protein–protein interaction network was built using STRING 11.0 (https://string-db.org/, accessed on 14 May 2020).

### 4.7. Statistical Analysis

All the experiments were carried out at least three times. The unpaired two-tailed Student’s *t*-test was used to calculate statistical significances and *p* < 0.05 were considered as statistically significant. Data visualization was performed using Prism 7.0 (GraphPad Software Inc., La Jolla, CA, USA). Numerical data were shown as means ± standard deviation (SD) if not otherwise specifically indicated and were derived from at least three independent experiments.

## Data Availability

Not available.

## Supplementary Materials

The following are available online, Figure S1: Representative spectra of mass spectrometry; Figure S2: dpECM proteins involved in (**A**) the pancreatic secretion pathway and (**B**) the insulin secretion pathway based on the KEGG pathway analysis; Table S1: dpECM proteins in adult porcine pancreata using the detergent-based method; Table S2: dpECM proteins in adult porcine pancreata using the detergent-free method; Table S3: dpECM proteins in young porcine pancreata using the detergent-free method; Table S4: GO terms of the proteins significantly regulated in adult or young dpECM.

## Sample Availability

Not available.
