# Supplementary material for: Proteomic and Bioinformatic Analysis of Decellularized Pancreatic Extracellular Matrices
Source: Molecules. 2021 Nov 8;26(21):6740. doi: 10.3390/molecules26216740 (PMC8588251; doi:10.3390/molecules26216740)
Supplement: Supplementary file 1 [file molecules-26-06740-s001.zip › Supplementary information dppECM 10-15-2021.pdf]

Supporting information

# Proteomics and Bioinformatic Analysis of Pancreatic tissues

*Ming Hu[1], Huanjing Bi[1], Deana Moffat[1], Margaret Blystone[1], Paul DeCostanza[1], Tchilabalo Alayi[2], Kaiming Ye[1,3], Yetrib Hathout[2], and Sha Jin[1,3]**

[1]Department of Biomedical Engineering, [2]School of Pharmacy & Pharmaceutical Sciences, [3]Center of Biomanufacturing for Regenerative Medicine.
Binghamton University, State University of New York (SUNY), Binghamton, New York 13902.
E-mail: sjin@binghamton.edu

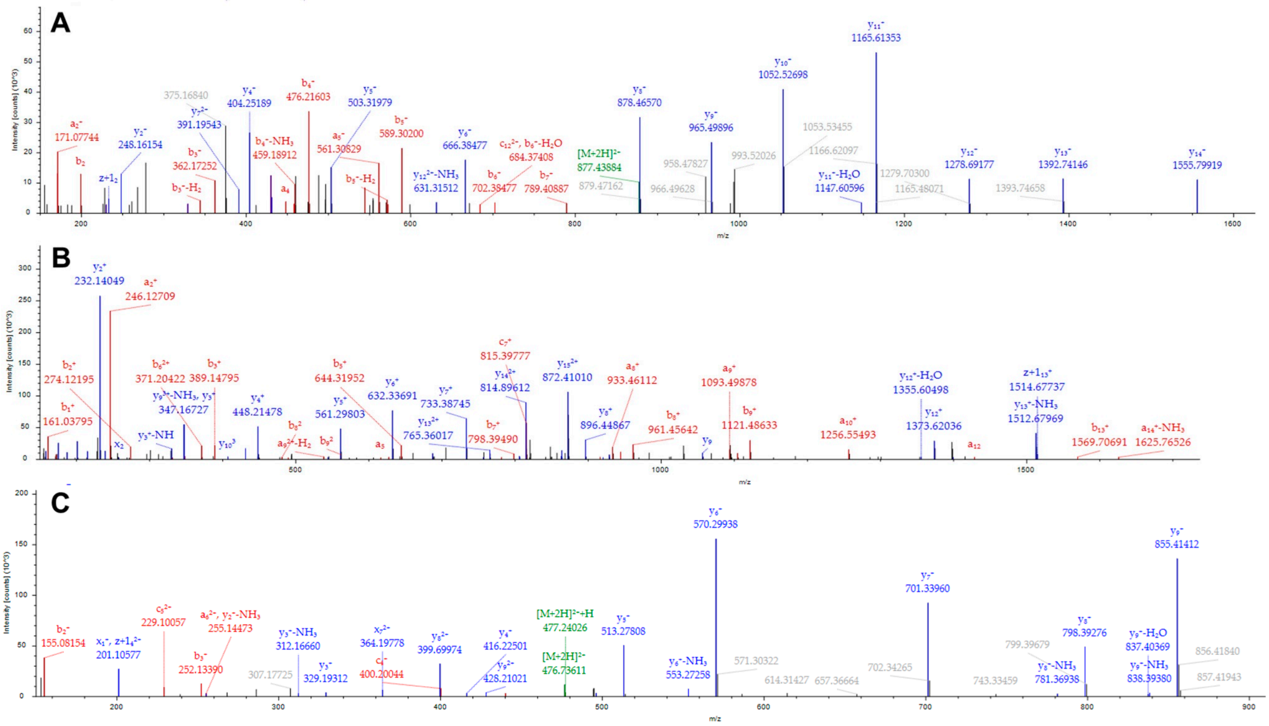


**Figure S1.** Representative spectra of mass spectrometry. (A) Laminin subunit alpha-2 isoform X1. Peptide TPYNILSSPDYVGVTK. (B) Fibrillin-1. Peptide CIDVRPGYCYTALTNGR. (C) Collagen alpha-1(I) chain. Peptide PGPMGPSGPR.

A

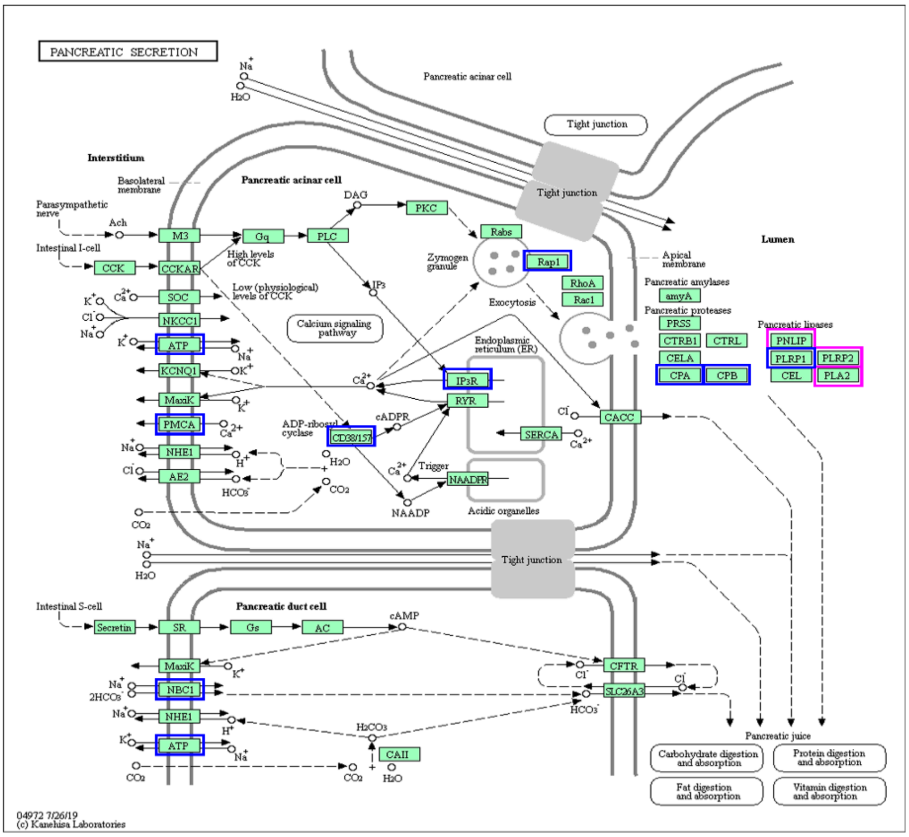


B

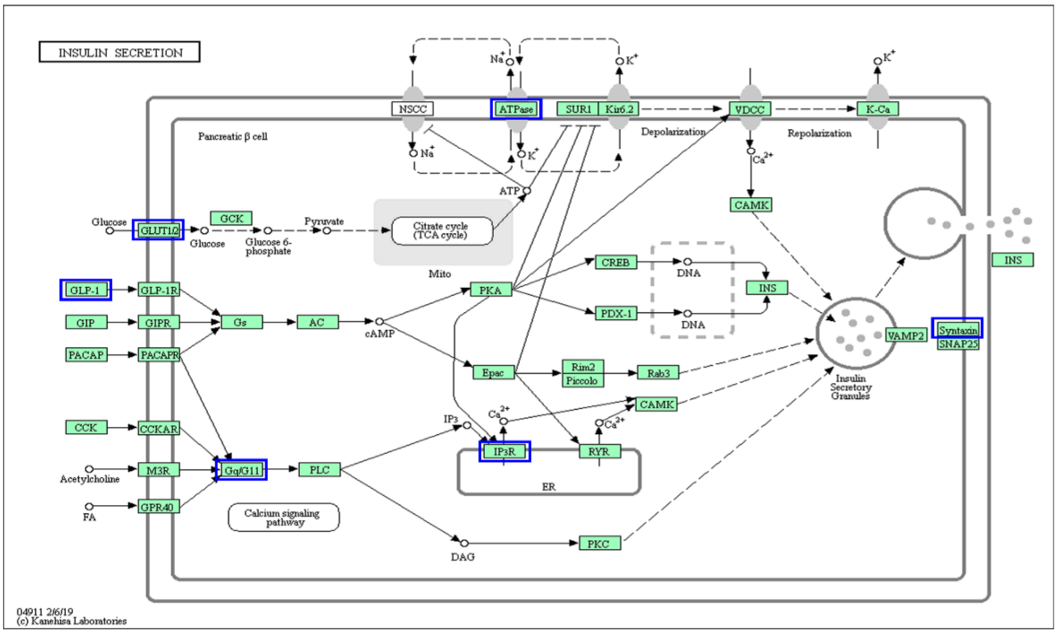


**Figure S2.** dpECM proteins involved in (A) the pancreatic secretion pathway and (B) the insulin secretion pathway based on the KEGG pathway analysis. Magenta boxed proteins are the proteins present in both adult and young dpECM, and blue boxed are proteins unique to young porcine dpECM.
